# A new chapter: hematopoietic stem cells are direct players in immunity

**DOI:** 10.1186/2045-3701-1-33

**Published:** 2011-10-06

**Authors:** Junke Zheng, Chun Song, Cheng Cheng Zhang

**Affiliations:** 1 Departments of Physiology and Developmental Biology, University of Texas Southwestern Medical Center, 5323 Harry Hines Boulevard, Dallas, Texas 75390, USA; 2 Shandong University and National New Drug R&D Center in Shandong, 44 West Wenhua Road, Shandong University, Jinan 250012, China; 3 Kemedicine Pharmaceutical LLC, 37 Science Center Road, Changping, Beijing 102206, China

**Keywords:** stem cell, hematopoietic stem cells, immunity, immunology, CD47, CD274, infection, inflammation, niche

## Abstract

Several lines of evidence support the hypothesis that hematopoietic stem cells (HSCs) directly interact with the immune system and have potential for immune privilege. Although the microenvironment or niche provides protection for HSCs from immune attack, HSCs are also capable of interacting with the immune system as signal "providers" and signal "receivers". On the one hand, HSCs display surface immune inhibitory molecules to evade the attack from the innate and adaptive immune systems; on the other hand, HSCs are capable of directly sensing the signals from the immune system through their surface receptors. Thus, HSCs are important direct players in the immune system.

## Introduction

The immune properties of pluripotent stem cells and tissue-specific stem cells have been attracting research interest. Based on the observation that embryonic stem (ES) cells express low levels of HLA class I and hardly any HLA class II proteins, it was initially suggested that ES cells are not recognized by the host immune system. However, well-designed experiments by Swijnenburg et al. clearly demonstrated that human ES cells were rejected upon transplantation into immune-competent recipient mice [1]. A novel study by Zhao et al. also showed that induced pluripotent stem (iPS) cells could trigger an immune response even in syngeneic recipients [2]. Therefore, it appears that pluripotent ES cells and iPS cells are not immune privileged. By contrast, some adult stem cells, such as mesenchymal and amnion stem cells are immune privileged to a certain extent. These cells are capable of avoiding rejection in intra- and even inter-species transplantation through the production of immunosuppressive molecules [1,3].

Hematopoietic stem cells (HSCs) are responsible for the daily production of all blood and immune cells in the body and have been widely used in transplantation to treat patients with leukemia, lymphoma, some solid cancers, and autoimmune diseases [4]. Although freshly isolated HSCs are known to have a very slim possibility of escape from immune rejection upon allogeneic transplantation, current evidence suggests that *in vivo *the HSC niche provides an immune-privileged site for HSCs. Moreover, HSCs per se also possess certain potential for immune privilege through the regulation of the expression of surface immune molecules. This short review, does not intend to cover the entire topic of the roles of HSCs in immunity; rather it seeks to provide an update of the recent progress toward the understanding of how HSCs survive immune-mediated elimination and how they contribute to immunity.

### HSCs in niches: immune privilege offered by regulatory T cells

*In vivo*, stromal cells and other cells form a complex microenvironment for HSCs that controls their multiple fates, including quiescence, apoptosis, and migration as well as the cell divisions that lead to formation either of daughter HSCs or of lineage-committed progenitors that are capable of limited proliferation. Currently, we know of the existence of several types of cells that form bone marrow HSC niches [5]. The endosteal HSC niche contains osteoblasts as the main supportive cell type for maintenance of hematopoiesis [6,7]. The vascular HSC niche is mainly composed of sinusoidal endothelial cells [8]. More recently, it was suggested that these two types of cells may establish a compound niche [9,10]. In addition, SDF-1 abundant reticular (CAR) cells [11], CD146-expressing subendothelial stromal [12], Nestin^+ ^mesenchymal stem cells [13], macrophages [14,15], and the sympathetic nervous system [16] have also been demonstrated to represent components of HSC niches [5].

A recent elegant study by Fujisaki et al. using high-resolution *in vivo *imaging demonstrated that regulatory T cells (Treg) colocalize with HSCs in the endosteal area in the bone marrow to protect HSCs from immune attack [17]. IL-10 produced by these Treg cells plays an essential role in this immune protection. This novel finding suggests that the HSC niche not only specifies an environment to control the cell fates of HSCs, but it may also provide an immune-privileged site for HSCs [17].

### CD47: innate immunity of HSCs

Whereas the niche offers protection for HSCs from immune attack, HSCs are capable of migrating in and out of the niche [18], which drastically increases the possibility that they will interact with the immune system. It is evident that HSCs outside of the niche apparently are capable of protecting themselves from innate macrophage phagocytosis.

CD47, also known as integrin-associated protein, binds to the signal regulatory protein alpha on macrophages and inhibits phagocytosis. Weissman's group showed that it is expressed on freshly isolated bone marrow HSCs at a relatively low level, suggesting that the possibility of the potential interaction between HSCs and the innate immune system within the stem cell niche is small. When HSCs are activated by potent inflammatory signals and mobilize into circulation, the CD47 level is dramatically upregulated on the surface of HSCs [19]. Similarly, CD47 is also upregulated on a variety types of blood cancer and solid cancer cells [20]. It was suggested that the increased expression of CD47 on the surface of mobilized HSCs and cancer cells protects these cells from phagocytosis [20].

### CD274: adaptive immunity of HSCs

In addition to evading the potential attack from the innate immune system, HSCs also have tricks that protect them against the adaptive immune system. CD274 (B7-H1 or PD-L1) is a member of the B7 family that is expressed on dendritic cells, activated immune cells, and parenchymal cells under certain condition and on cells in immune-privileged sites such as eyes and placenta where it inhibits T cell or innate activation [21,22]. CD274 is also selectively expressed by various cellular components in the tumor microenvironment, where it inhibits tumor-specific T-cell immunity by inducing T cell apoptosis and delaying rejection [21].

We recently provided evidence demonstrating that HSCs possess the ability to regulate their surface expression of CD274 in order to evade the rejection by the acquired immune system [23]. We showed that CD274 is expressed on freshly isolated bone marrow HSCs at a low level *in vivo *[23]. These are similar to the scenario of CD47. Surprisingly, after *in vitro *culture, HSCs upregulate the surface expression of CD274 10-fold, which efficiently inhibits host T cell proliferation upon allograft transplantation [23]. These observations clearly indicate that *ex vivo *culture significantly modulates the immunogenicity of stem cells. Therefore, in striking contrast to pluripotent stem cells, HSCs have modulatable immune privilege that can overcome allogeneic immune barrier, and HSCs can directly participate in adaptive immunity.

Fiorina et al. demonstrated that CD274 is upregulated on mouse splenic Lin^-^Kit^+ ^hematopoietic cells after the treatment of an antagonist of chemokine CXCR4 [24], suggesting that the CD274 level on phenotypic hematopoietic progenitors can be increased upon mobilization. Whether the expression of CD274 on primitive and functional HSCs or blood cancer cells can be physiologically regulated *in vivo *and its biological significance warrants further investigation. Moreover, the future identification of additional immune molecules whose alterations (either up or down) can regulate allograft acceptance will enable the complete resolution of the issue of immune rejection in allogeneic transplantation.

### HSC activation by infection

While the existence of niche and the expression of immune inhibitors on HSCs enable HSCs to evade immune attack, it is also clear that HSCs can directly respond to pathogen-specific infection through systematic cytokine stimulation from both innate and adaptive immune signals. Several classes of signaling receptors expressed on HSCs that bind to cytokines or infectious ligands directly participate in the infection response: IFN receptors [25-27], TNF receptor [28-30], and Toll-like receptors (TLR) [31-33] (see review by Goodell's group [34]). In general, the infection or inflammatory signals activate HSCs and chronically lead to accelerated differentiation at the expense of loss of HSC potency [27]. The differentiation produces immune effector cells that counteract the initial infection. By contrast, the aberrant IFN and TNF signaling are associated with myelodysplastic syndrome and bone marrow failure [34]. We predict that additional immune-related surface signaling receptors also regulate the cell fates of HSCs. For example, the identification of the receptors for Angiopoietin-like proteins (Angptls) [35-38] will enable us to elucidate the effects of inflammatory Angptls on HSCs (unpublished data).

### Model of the interplay between HSCs and the immune system

**Figure 1:**
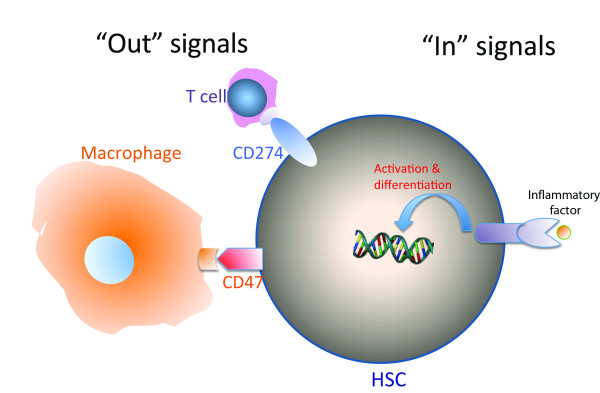
**A model of the interplay between HSCs and the immune system**. HSCs express surface immune molecules for "in" signaling and "out" signaling that directly dialog with the immune system. Whereas the "out" signaling, mediated by surface molecules such as CD47 and CD274, inhibits attack from the innate immunity and adaptive immunity responses, respectively, the stimulatory "in" signaling from inflammation activates HSCs and induces differentiation through surface receptors including TLR, TNFR, IFNR, and others.

Based on these recent progress, we propose a model for the interaction between HSCs and the immune system. Within the niche, HSCs are protected by Treg cells from potential immune attack. Outside the niche, HSCs are capable of directly interacting with the immune system through surface immune molecules for "out" signaling and "in" signaling (Figure 1).

The "out" signaling is mediated by factors such as CD47 and CD274 that inhibit attack from the innate immunity and adaptive immunity responses, respectively. Based on the results discussed here, we hypothesize that homeostatic HSCs express low levels of surface immune suppressors, and the levels of these suppressors can be induced by stress or immune signals. These immune suppressors may thus modulate HSC immunogenicity and, therefore, contribute to the "immune privilege" of HSCs. This regulatable "immune privilege" should be advantageous to HSCs as it should allow these important stem cells to rapidly adjust to altered environmental conditions or protect them from excessive immune activation/inflammation or potential autoimmune disorders.

The stimulatory "in" signaling from inflammation activates HSCs and induces differentiation through surface receptors including TLR, TNFR, IFNR, and others. This activating "in" signaling should be counter-balanced by the environmental cues within the stem cell niche, which maintain the quiescence and stemness of HSCs. The continuing investigation of this area may open up a new scientific field - the immunology of stem cells. We speculate that a common mechanism exists for regulation of immune signals in some other types of stem cells. It will be interesting to study the immunology of stem cells by investigating the roles of surface immune molecules and receptors on ES cells, iPS cells, other adult stem cells, and cancer stem cells.

### Open questions

The following questions must be addressed to provide new insights into the understanding of immunology of stem cells:

1) What surface immune molecules in addition to CD47 and CD274 are expressed on HSCs and other stem cells that modulate the immunogenicity of stem cells?

2) Do HSCs express immune stimulatory molecules? Probably yes. If so, what are their functions in modulating other immune cells?

3) How is the expression of immune surface molecules regulated *in vivo*? What is the mechanism for the potential upregulation of immune suppressors and downregulation of immune stimulators? Do surface immune molecules change their expression on HSCs upon mobilization, infection, and in extramedullary organs?

4) HSCs can interact with immune system as signal providers ("out" signaling) and as signal receivers ("in" signaling). What is the connection between the "in" signaling and "out" signaling? Can "in" signaling modulate the type and magnitude of "out" signaling and vice versa? The study of CD274 may provide more insight into this question. For example, CD274 is not only a ligand for PD-1 and CD80 (thus providing "out" signaling) but is also a counter-receptor of PD-1 that may deliver the reverse "in" signaling.

5) Can HSCs sense signals from the immune system to attenuate their activation? Does this help maintain their stemness?

6) Do human and mouse stem cells have similar immunology? For example, do CD274 and other surface immune molecules play similar roles in both species?

7) Does the aberrant immune property of stem cells cause diseases?

8) What are the expression and functions of surface immune molecules and receptors on other stem cells including ES cells, iPS cells, other tissue specific stem cells, and cancer stem cells?

## Conclusion

Recent evidence suggests that HSCs are immune privileged within the *in vivo *bone marrow niche. Nevertheless, HSCs are also capable of interacting with the immune system as signal "providers" and signal "receivers". As signal providers, HSCs display surface immune inhibitory molecules to suppress the innate and adaptive immune systems. As signal receivers, HSCs directly sense systematic immune signals through their surface receptors and change their cell fates in response to the altered immune environment. HSCs thus directly interact with the immune system. The data discussed here mark the start of a series of investigations that may open up a new field - the immunology of stem cells.

## List of abbreviations

Angptls: angiopoietin-like proteins; ES cells: embryonic stem cells; HSCs: hematopoietic stem cells; iPS cells: induced pluripotent stem cells; TLR: toll-like receptor; Treg: regulatory T cells.

## Competing interests

The authors declare that they have no competing interests.

## Authors' contributions

CCZ, JZ, and CS wrote the manuscript. All authors read and approved the final manuscript.
